# Cystic Endometriosis in a Huge Degenerated Subserous Leiomyoma Mimicking Bilateral Multicystic Endometriomas in an Infertile Woman with Diminished Ovarian Reserve: A Rare Endometriotic Implantation

**DOI:** 10.1155/2016/2713943

**Published:** 2016-02-29

**Authors:** Safak Hatirnaz, Sabri Colak, Abdulkadir Reis

**Affiliations:** ^1^IVF Center, Konak Hastanesi, 41000 Kocaeli, Turkey; ^2^Department of Obstetrics and Gynecology, Kanuni Egitim ve Arastirma Hastanesi, 61100 Trabzon, Turkey; ^3^Reis Pathology Laboratuari, 61100 Trabzon, Turkey

## Abstract

Uterine leiomyomas are the most common pelvic tumor in women. Leiomyoma can show atypical locations and degenerations and may not be easily differentiated from adnexal masses. Uterine leiomyoma can undergo cystic degeneration and is said to be found in 4% of all types of degenerations. The commonest type of degeneration is hyaline seen in 60% of patients. Usually uterine leiomyoma does not present as clinical and radiological diagnostic challenge. However, when leiomyoma undergoes massive cystic degeneration they may become clinical and radiological diagnostic dilemmas. The MRI showed a huge cystic mass protruding up to the pelvis not differentiated from bilateral endometriomas and accompanying subserous myomas. Surgery revealed that the mass is not bilateral endometriomas but a huge pedunculated leiomyoma with cystic degeneration and cystic endometriosis. Endometriosis is a troubling gynecologic condition occurring in 10% to 15% of women of reproductive age and is associated with fertility problems. As a peritoneal disease, the locations of endometriotic lesions are predominantly the ovaries (96.4%), followed by the soft tissue (2.8%), gastrointestinal tract (0.3%), and urinary tract (0.2%) and other rare locations. The presented case is multiple sized cystic endometriosis (endometriomas) located in a huge pedunculated subserous leiomyoma in an infertile woman having a history of laparoscopic bilateral endometrioma surgery.* Conclusion*. To our knowledge, this is the first reported case for endometriotic cysts (endometriomas) located in a huge cystic degenerated leiomyoma. PubMed search revealed no report concerning endometriotic implantation in the leiomyomas.

## 1. Introduction

Leiomyoma is the most common benign gynecological tumor seen in women [1, 2].

Leiomyomas are classified as submucous, intramural, and subserous according to their origin and the symptoms and signs differ according to the location [1, 2]. Leiomyoma degeneration may be misdiagnosed as adnexal masses or malignancy. Hyaline degeneration is the most common leiomyoma degeneration while cystic degeneration and calcific degeneration can be seen in leiomyomas [1, 2]. Leiomyoma degenerations are mostly symptomatic but asymptomatic degenerations can be seen in some cases. Some subserous leiomyomas are pedunculated and symptoms can be seen due to torsion of the myoma pedicle.

Endometriosis is a common gynecologic disease affecting 10% to 15% of reproductive age women and is associated with various degrees of fertility problems [3]. Endometriosis is defined as the presence of endometrial glands and stroma at extrauterine sites.

Endometriosis is a common, benign, chronic, estrogen-dependent disorder. It can be associated with many distressing and debilitating symptoms, such as pelvic pain, severe dysmenorrhea, dyspareunia, and infertility, or it may be asymptomatic and incidentally discovered at laparoscopy or exploratory surgery.

These ectopic endometrial implants are usually located in the pelvis but can occur nearly anywhere in the body. The common locations of endometriosis are the ovaries, fallopian tubes, pelvic peritoneum, and uterosacral ligaments (also called pelvic site), while the atypical sites of endometriosis include the gastrointestinal tract, urinary tract, soft tissues, and chest, nose, and incisional sites (also called extrapelvic site) [4]. Endometriosis lesions can be classified as ovarian, extraovarian, or mixed. The predominant location of endometriosis was the ovaries (96.4%), followed by the soft tissue (2.8%), gastrointestinal tract (0.3%), and urinary tract (0.2%) [5].

Endometriosis at locations outside the pelvis is explained by dissemination of endometrial cells or tissue through lymphatics and blood vessels.

The coelomic metaplasia theory proposes that the coelomic (peritoneal) cavity contains undifferentiated cells or cells capable of dedifferentiating into endometrial tissue. This theory is based upon embryologic studies demonstrating that all pelvic organs, including the endometrium, are derived from cells lining the coelomic cavity.

One hypothesis is that secretion of various cytokines by endometriosis implants and inflammatory cells into the peritoneal cavity leads to proliferation of implants, recruitment of capillaries (e.g., by vascular endothelial growth factor), and chemoattraction of leukocytes to these foci of peritoneal inflammation [6]. Oxidative stress may be another component of the inflammatory reaction [7]. Thus, the immune system may play a role in determining who will develop endometriosis, as well as the extent and clinical manifestation of the disease [8, 9].

Endometriotic implantation either by aspiration needle inoculation or by hypothetical invagination of endometriomas into the degenerated leiomyoma is an extreme rarity.

## 2. Case

46-year-old woman with 8 years of primary infertility was admitted to Clinart International Hospital for bilateral multicystic complex adnexal masses diagnosed 4 years ago as bilateral endometriomas. She had 7 IVF attempts and only in 3 cycles; embryo could be transferred with no clinical outcomes. She had a history of laparoscopic endometrioma surgery 8 years ago and endometriomas on both ovaries were aspirated and their capsules were excised together with the laparoscopic excision of a subserous myoma. Ca 12-5 and Ca 19-9 values had been high before the laparoscopic surgery. She had no other medical or surgical history and all biochemical serological and hormonal parameters were normal other than high Ca 12-5 and Ca 19-9 which supported the diagnosis of endometrioma.

MRI investigation was carried out 2 years ago and bilateral endometriomas were reported without any radiological signs of malignancy. Patient was informed about the possible risks of malignant transformation of the adnexal masses at all times; she was admitted for fertility reasons but surgical intervention was rejected. All attempts for fertility were failed due to either the absence or diminished ovarian response to ovarian hyperstimulation by gonadotropins. Some cycles were monitored naturally with no collected oocytes. It was not easy to differentiate the follicular response by ultrasound and all oocytes retrieved from the follicles were aspirated through the endometriomas and endometriotic fluid was seen in the aspiration tubes. Oocyte retrieval (OR) by bypassing the endometriomas was not possible technically because the masses on both sides were huge and multilobulated and ovaries could not be discriminated easily. In two cycles the endometriomas were aspirated to reduce the size of the mass and to obtain oocytes easily under the suppressive antibiotherapy.

**Figure 1:**
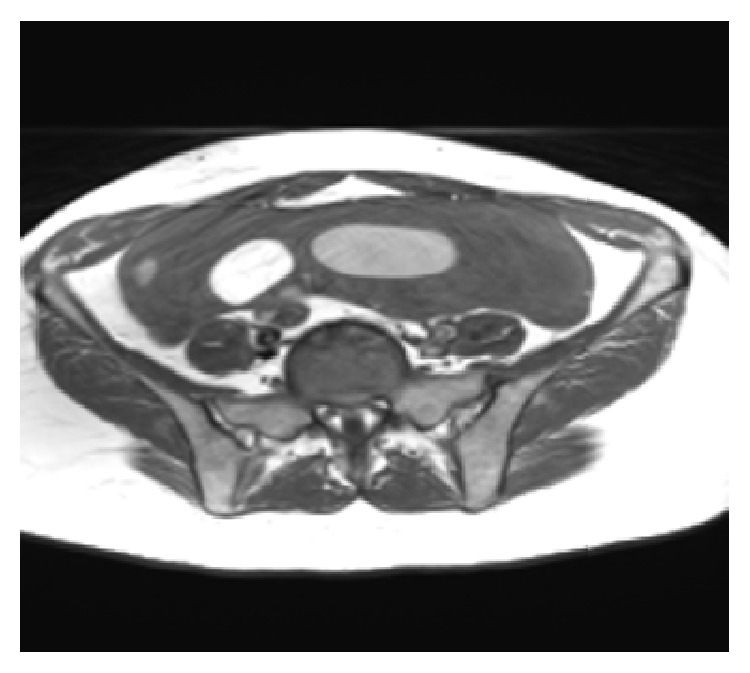
MRI picture of the patient.

**Figure 2:**
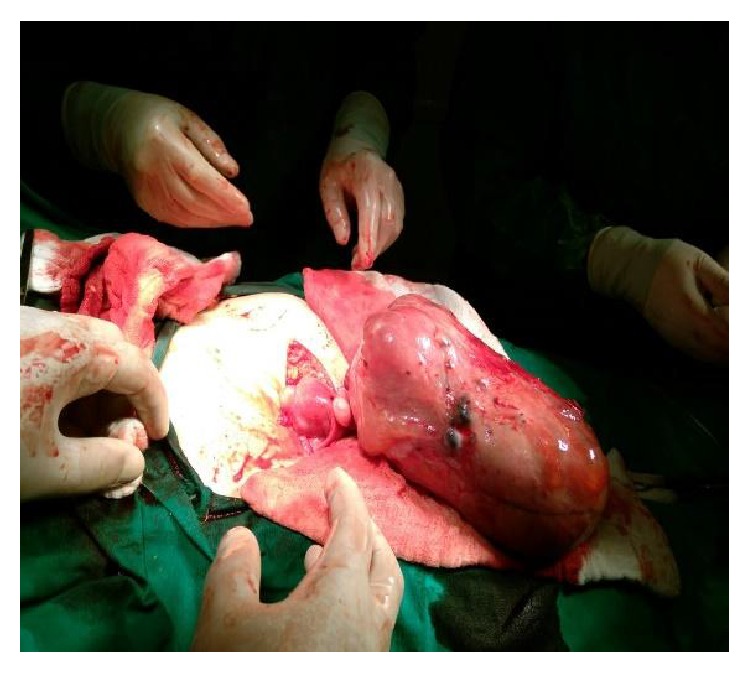
Pedunculated huge mass originating the anterior surface of the uterus.

**Figure 3 fig3:**
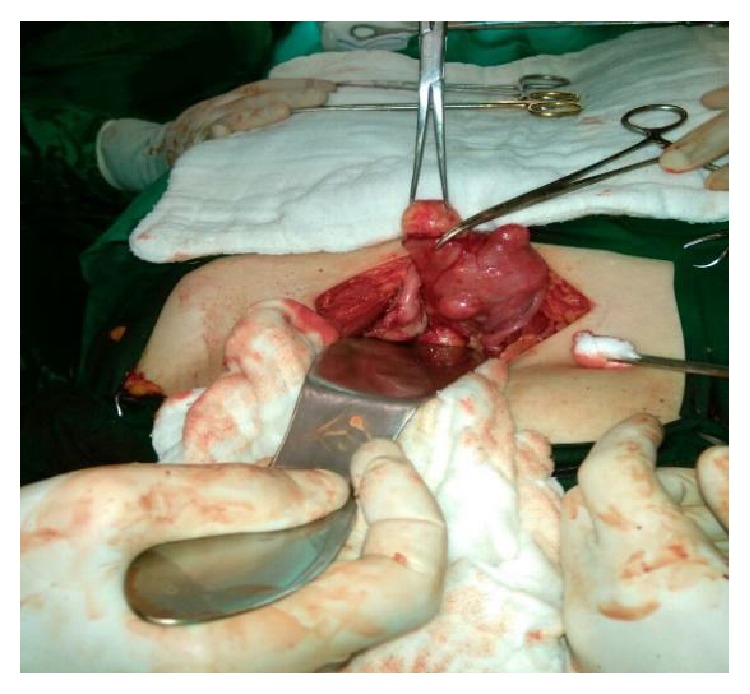
Both tubas and ovaries seen (no endometrioma or endometriotic lesions).

**Figure 4 fig4:**
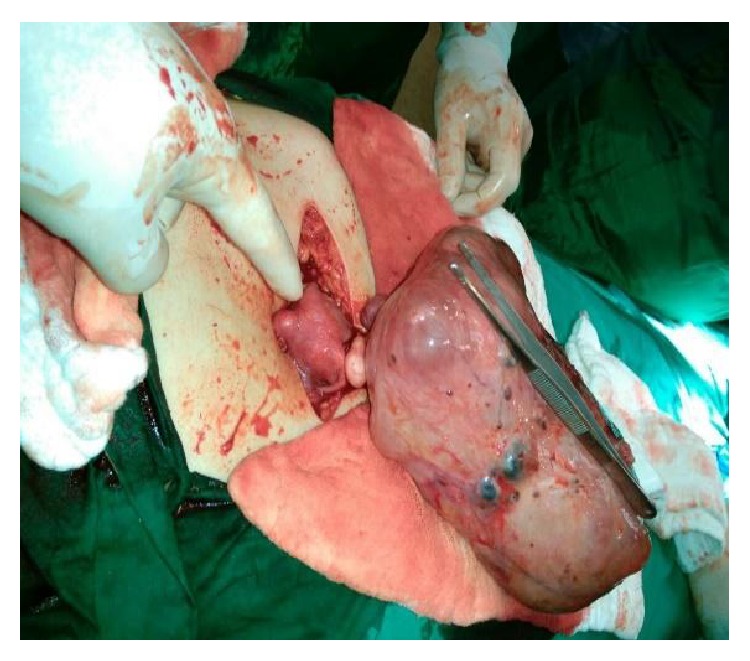
Blue discoloration-lesions on the mass.

**Figure 5:**
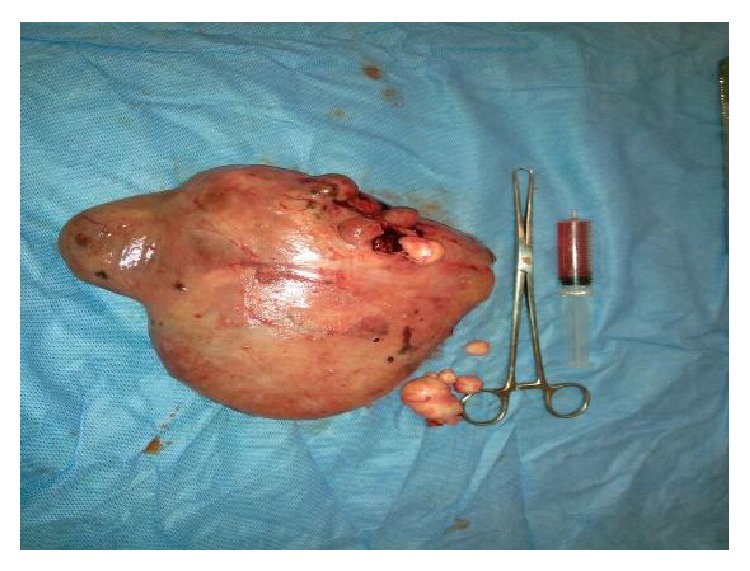
Extirpated leiomyomas.

She decided to have the surgical operation and admitted for surgery at 31.03.2015. Pelvic MRI (Figure 1), Ca 12-5, Ca 19-9, and all preoperative tests were carried out same day and open surgery under general anesthesia was planned and carried out at 01 04 2015 by a Pfannenstiel incision. The mass could easily be seen from the abdominal wall before making the incision. A huge lobulated smooth surface mass of 22 × 18 × 10 cm in size was seen by opening the peritoneum and the first impression of the surgical team was malignancy and peritoneal washing was aspirated for cytological evaluation in Figures 2 [Fig fig3] [Fig fig4] and 5.

The mass was well vascularised and adherent and a very thick vascularised adhesion was separated by electrocautery and sutured. Then the mass was taken out and it was a great surprise for all the team that the huge mass was originating from the anterior surface of the uterus just below the right corn and was evaluated as pedunculated degenerated leiomyoma with a thick pedicle.

Thorough search of the uterus and ovaries revealed 4 subserous leiomyomas of different sizes and both ovaries were free from any endometriotic lesions and left fallopian tube was adherent to the posterior of the uterus. Left ovary was found atrophic and right ovary was found smaller than normal. No endometriomas or endometriotic lesions were seen on the ovaries, tubes, and the uterus. Multiple leiomyomas at different size were seen on the surface of the uterus and uterus myomatosis was diagnosed intraoperatively.

She was supposed to have bilateral endometriomas for long time and the mass was misinterpreted as endometrioma both clinically and radiologically. The reason why the mass was interpreted as multilobulated bilateral endometrioma was due to the chocolate coloured aspiration materials seen in oocyte pick up procedures and also high Ca 12-5 and Ca 19-9 values. The mass was excised from the uterus by sharp dissection and the uterus was sutured with 1/0 Vicryl. Macroscopical evaluation of the mass at the operating theatre revealed multiple small nodules and a small leiomyoma seen on the surface close to the pedicle of the mass. Intraoperative evaluation showed multiple blue discolorations on the mass and no solid component was seen within the excised mass. The other leiomyomas were excised and sutured and the adherent left side tube was released from the uterus by sharp dissection and following hemostasis, irrigation, and aspiration, abdominal wall was closed layer by layer. Total amount of blood loss was 150 mL and the duration of operation was one and a half hours and no surgical and anesthesiological complications were observed. The pathology lab was informed verbally about the case and detailed information was given and aspirated fluid and the extirpated leiomyomas were sent to the pathology laboratory for cytological and pathological evaluation.

Intraoperative and postoperative macroscopic figures are shown in Figures 2–5.

## 3. Pathology Report

### 3.1. Cytological Diagnosis

No malignant cells were elucidated from the peritoneal aspiration fluid.

### 3.2. Macroscopy

**Figure 6:**
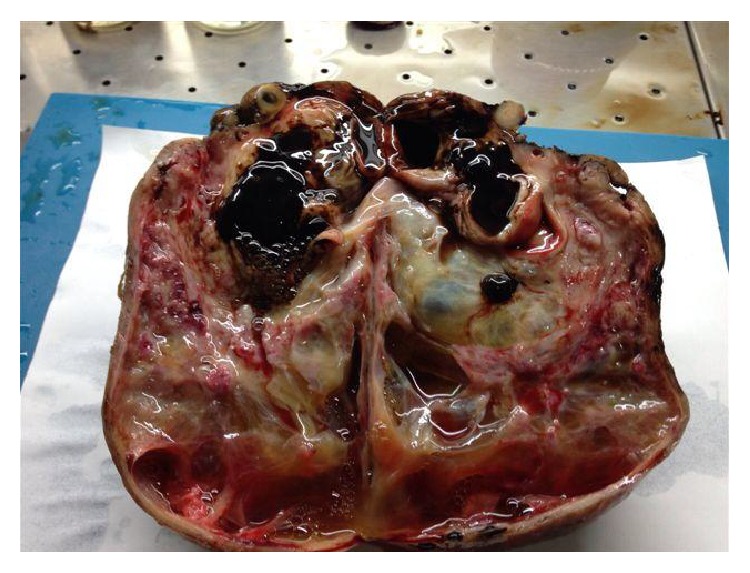
Macroscopic figure; endometrioma in the pseudocystic degenerated mass.

A mass weighed 1285 grams and 18 × 18 × 8 cm in size with smooth surface was evaluated and 10 × 7 cm surgical excision site was visualised together with nodular leiomyomas (one separated from the mass and another in size of 4 cm). The mass was excised sagittally and first impression of the mass was cystic degenerated leiomyoma with mucoid melting plus multiloculated cystic mass (12 × 8 × 7 cm in size) filled with sticky dark brown liquefied bloody fluid (chocolate cyst) (Figure 6).

### 3.3. Microscopy

**Figure 7:**
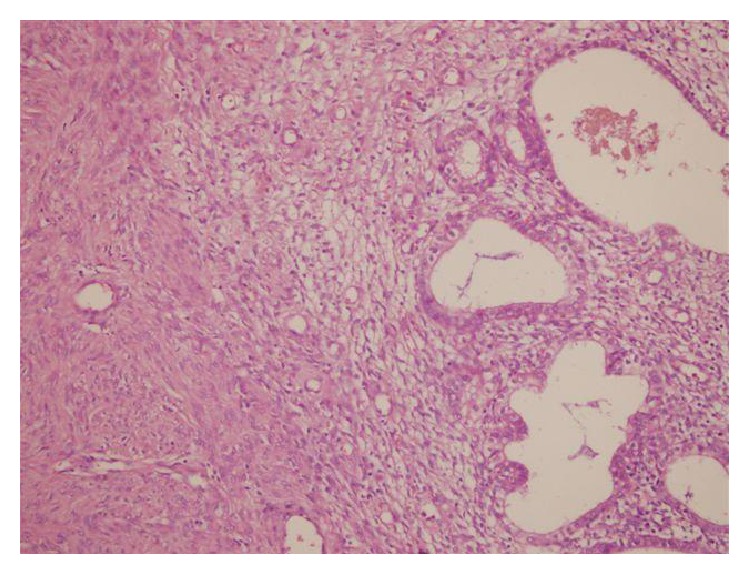
Endometriotic focus having endometrial stroma and glands in leiomyoma.

**Figure 8:**
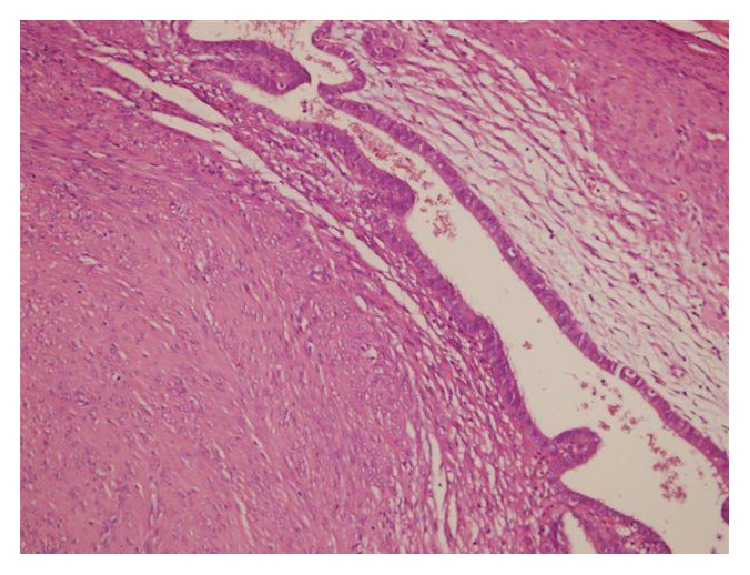
Cystic endometrial epithelial lining in leiomyoma (HE ×200).

Endometrial glandular epithelial linings were found in the wall of blood filled wide cystic structures in the dissections of degenerated leiomyoma and multiple foci of endometriosis and wide range of old blood (hemosiderophages) were found in the leiomyoma (Figures 7 and 8).

Cystic endometriosis in degenerated leiomyoma (microscopic Figures 7 and 8).

### 3.4. Immune Histochemical Study (IHC)

**Figure 9:**
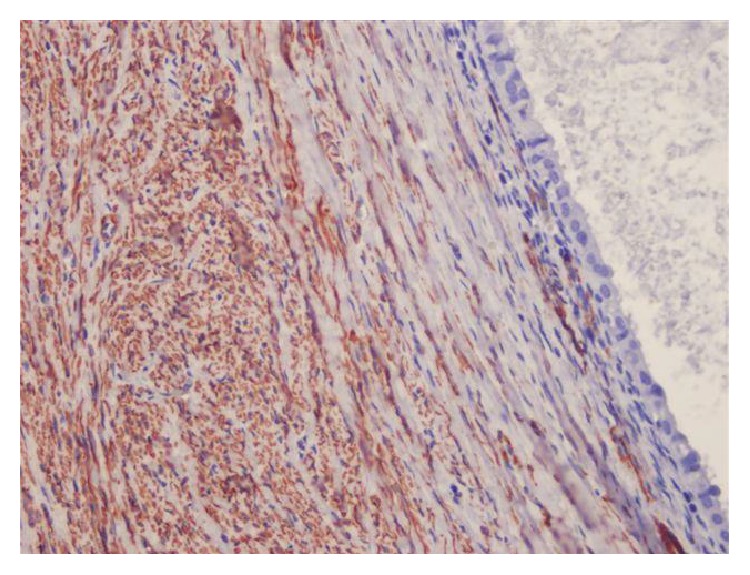
Actin immunoreactivity in neoplastic smooth muscle cells (IHC ×200).

**Figure 10:**
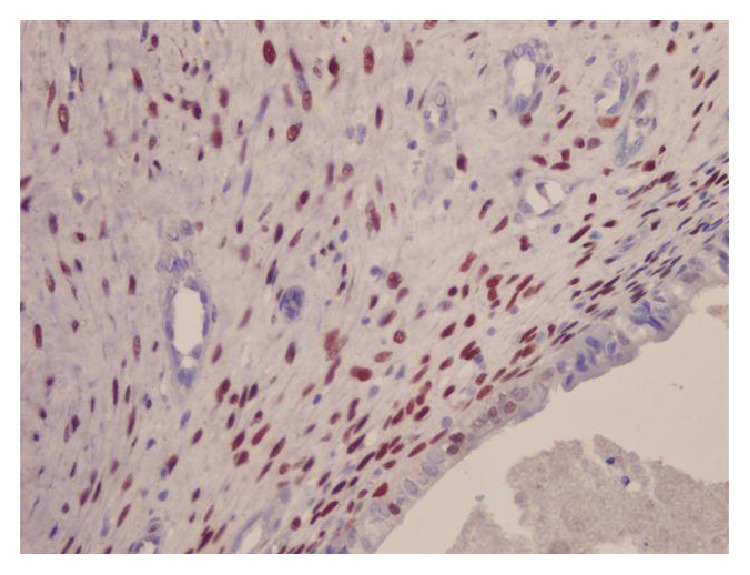
Nuclear progesterone receptor immunoreactivity in both smooth muscle cells and endometrial epithelial cells (IHC ×400).

Immunohistochemical tests including actin, CK7, CK20, oestrogen receptor, and progesterone receptor were studied (Figures 9 and 10).

### 3.5. Pathological Diagnosis

Cystic endometriosis in a huge degenerated leiomyoma originates from anterior uterine serosa.

## 4. Discussion

Uterine leiomyomas affect 20%–30% of women older than 35 years and exhibit various growth patterns and can be symptomatic as abnormal bleeding dysmenorrhea pelvic pain and pressure related symptoms to neighboring organs [2, 10]. Subserous leiomyomas are relatively asymptomatic until they become huge masses. Smooth muscle tumors can undergo secondary changes like hyaline, cystic, myxoid, and red degenerations depending on their location or size [11, 12] mostly due to improper blood supply of such hungry tumors.

Cystic degeneration may be a sequela of edema and reported to be seen in 4% of all leiomyomas [11, 12]. The wall and septum of the cystic leiomyomas are thick as in this case [2, 5, 11, 12]. A pedunculated leiomyoma can grow into the broad ligament and become parasitic and can detach from the uterus and be nourished by the retroperitoneal blood supply [5, 12].

Extrauterine leiomyomas are rare and these benign tumors originating from smooth muscle cells usually arise in the genitourinary tract (in the vulva, ovaries, urethra, and urinary bladder) but may arise in nearly any anatomic site. In addition, unusual growth patterns may be seen, including benign metastasizing leiomyoma, disseminated peritoneal leiomyomatosis, intravenous leiomyomatosis, parasitic leiomyoma, and retroperitoneal growth. However, some extrauterine leiomyomas may mimic malignancies, and serious diagnostic errors may result. The most useful modalities for detecting extrauterine leiomyomas are ultrasonography, computed tomography, and magnetic resonance (MR) imaging. The superb contrast resolution and multiplanar capabilities of MR imaging make it particularly valuable for characterizing these tumors, which usually show low signal intensity similar to that of smooth muscle on T2-weighted images. The radiologist's recognition of this and other characteristic features may help steer the clinician toward timely, appropriate management and away from unnecessary, potentially harmful treatment [13].

Pedunculated cystic leiomyomas should be considered in the differential diagnosis of multiloculated cystic adnexal masses since sonographic and CT findings cannot differentiate these tumors from ovarian malignancy [11]. Typical appearances of uterine leiomyoma at magnetic resonance (MR) imaging are well established, and diagnosis is usually easy.

However, cases that are extremely difficult to differentiate from other conditions are occasionally encountered. To understand the wide spectrum of MR imaging findings, such unusual appearances can be classified into three categories: degeneration and other histopathologic findings, specific types of unusual leiomyomas, and unusual growth patterns. The common types of degeneration are hyaline (>60% of cases), cystic (approximately 4%), myxoid, and red. Edema is not a phenomenon of degeneration but is a common histopathologic finding (approximately 50% of cases). Hemorrhage, necrosis, and calcification (approximately 4% of cases) may also be observed. Specific types of unusual leiomyomas include lipoleiomyoma and myxoid leiomyoma, which may have MR imaging features characteristic enough to allow differentiation from other gynecologic and nongynecologic diseases. Intravenous leiomyomatosis, metastasizing leiomyoma, diffuse leiomyomatosis, and peritoneal disseminated leiomyomatosis represent unusual growth patterns; other unusual growth patterns are retroperitoneal growth, parasitic growth, and the pattern that may occur in cervical leiomyoma. Because leiomyomas are the most common gynecologic tumors and are exclusively benign, it is important to be familiar with the variety of MR imaging appearances of uterine leiomyomas to distinguish them from other significant diseases [14].

Endometrioma located mostly in the ovaries is a benign cystic tumor formed by the invagination of the cortex of the ovary. Invagination of the endometriotic lesions is the main phenomenon for the formation of endometriotic masses and this process is also seen in deep infiltrating endometriosis, especially in lower intestinal endometriosis lesions. Endometriotic lesions make the neighboring organs and tissues adhere to ovary to make conglomerated mass lesions as its nature.

The implantation of the endometriosis into the degenerated myoma may be due to the inoculation of endometriotic cells by follicular aspirations during OR but we cannot explain the myth of the absence of endometriotic implants on pelvic organs or any remnants of the bilateral endometriomas on both ovaries since the patient had a history of laparoscopic surgery for bilateral endometriomas together with laparoscopic subserous leiomyoma extirpation. The invagination of the endometriomas from the needle insertion sites into the degenerated myoma might be the reason but this could be a probable hypothesis not the fact because the capsule of the leiomyoma was very thick.

However the pedicle insertion site to the mass was very thin compared to the rest of the mass and one small leiomyoma and 3-4 soft nodules were seen at the insertion site. The biggest cystic endometriotic lesion was close to the edge of the capsule and that site was thin too.

In one of the rarest disease leiomyomatosis peritonealis disseminata (LPD) endometriosis can coexist in some lesions and this may be explained due to submesothelial multipotent stem cells also called secondary Müllerian system and endometrium originates from the same epithelium; thus endometriosis can coexist with disseminated leiomyoma [12].

## 5. Conclusion

Degenerated pedunculated leiomyomas can be misdiagnosed as adnexal masses both clinically and radiologically. Endometriosis can be seen in rare locations. Cystic endometriosis located in a huge pedunculated degenerated leiomyoma is an extremely rare endometriotic implantation and to the best of our knowledge this is the first case reported.

## Abbreviations

HE: Hematoxilen Hematoxylin eosin
IHC: ImmunehistochemistryImmunohistochemistry.
